# Mixing methods: How listening to researchers, healthcare workers, and community members can improve the design of studies testing medical innovations for Neglected Tropical Diseases

**DOI:** 10.1371/journal.pntd.0013547

**Published:** 2025-09-30

**Authors:** Astrid C. Erber, Anthony Ablordey, Adamu Addissie, Evans K. Ahortor, Doris Burtscher, Esther M. Mukooza, Richard Creswell, Ben Lambert, Piero L. Olliaro

**Affiliations:** 1 Department of Epidemiology, Centre for Public Health, Medical University of Vienna, Vienna, Austria; 2 Infectious Diseases Data Observatory (IDDO), Oxford, United Kingdom; 3 Department of Bacteriology, Noguchi Memorial Institute for Medical Research, University of Ghana, Legon, GhanaGhana; 4 School of Public Health, College of Health Sciences, Addis Ababa University, Addis Ababa, Ethiopia; 5 School of Pharmacy and Pharmaceutical Science, Cardiff University, Cardiff, Wales, United Kingdom; 6 Vienna Evaluation Unit/Anthropology, Médecins Sans Frontières, Vienna, Austria; 7 Department of Operational Research, Médecins Sans Frontières, Manzini, Eswatini,; 8 Department of Computer Science, University of Oxford, Oxford, United Kingdom; 9 Department of Statistics, University of Oxford, Oxford, United Kingdom; 10 International Severe Acute Respiratory and Emerging Infection Consortium (ISARIC), Pandemic Sciences Institute, University of Oxford, Oxford, United Kingdom; Oswaldo Cruz Foundation: Fundacao Oswaldo Cruz, BRAZIL

## Introduction

The stakes of evaluating medical innovations for Neglected Tropical Diseases (NTDs) and other infectious diseases of poverty are especially high. It is crucial to design trials that yield meaningful outcomes for diseases that burden specific vulnerable populations. Due to inadequate investments into research and development for diagnostics, treatments, and vaccines, opportunities to test new products are few and far between. Study designs should be tailored to addressing the key needs of users (patients) and providers (such as healthcare workers [HCWs]), so as to ensure swift uptake and implementation of research outputs.

There is often considerable uncertainty or ignorance regarding studies’ key aspects such as

- Understanding of the context: *In what context are innovations required to work, what could a typical setting look like?*
- Assessment of evidence: *Are there sufficient data to inform decisions on study design and the target population, and to inform parameters and assumptions? How could such studies be initiated and run in more challenging environments?*
- Understanding and acceptability of the study procedures: *Do members of the studies’ ‘target’ communities understand and accept study procedures related to recruitment and informed consent, the intervention and study outcomes? And, related to this, how do they feel about participation, what is their experience with previously conducted studies that might influence perception and acceptance?*

To answer these questions, the exploratory nature of small and pragmatic qualitative studies is well-suited to complement diagnostic test accuracy (DTA) studies or randomized controlled trials (RCTs). Study designs can be optimized by incorporating the combined input of researchers, HCWs, and users (patients or communities) upfront—which is rarely done for NTDs. Embedded mixed methods designs, with the qualitative component tightly integrated within the main or “parent” study, can address the above-mentioned key aspects and also provide opportunities for highly participatory research designs.

Here, we provide some illustrative examples of such studies that have been applied to NTDs in order to address the aspects above, and discuss their methodological approaches.

## Examples

In a study published earlier [1], we evaluated a loop-medicated isothermal amplification (LAMP) protocol independent of electricity supply for the diagnosis of Buruli Ulcer (BU). For this study, we also elicited views from researchers, HCWs, and community-based healthcare volunteers via interviews and focus group discussions on the diagnostic and treatment pathway at a small health clinic in a BU-endemic area in Ghana’s Eastern region, thereby characterizing a potential target setting. Findings included barriers such as costs and lack of transport options that patients had to meet when seeking treatment, and supported the potential usefulness of LAMP in this setting [2].

Approaches addressing a lack of data include consultations of experts, such as researchers or clinicians, in order to inform models for analysis. Pioneering work has been conducted, for example, by Joseph and colleagues on the diagnosis of strongyloides infections [3]. When incorporating such expert knowledge into statistical analysis, it may be attractive to employ Bayesian methods, as they can readily admit expert knowledge via *priors.* Priors are probability distributions which express beliefs about parameter values (e.g., the sensitivity or specificity of a diagnostic test) before new evidence is observed. As new data become available, Bayesian methods update these beliefs in a statistically rigorous way which simultaneously accounts for prior beliefs and the evidence contained in the data. A variety of different approaches can be used to encode expert beliefs into prior distributions, and these do not require that the experts themselves (whose expertise may lie in other fields besides statistics) have extensive statistical knowledge [4]. Such approaches could take the form of a participatory and iterative dialogue between disease experts and statisticians, and also help to make decisions surrounding data and data selection, parameters, and assumptions more explicit and accessible to readers. A limited number of studies have demonstrated this for RCTs [5] or DTAs [3]. Using expert elicitation approaches can also address a lack of data (for example, disease prevalence or incidence rates, which might be under-reported or not available), to refine model parameters and assumptions, and could provide support with other decisions, such as the selection of suitable datasets for meta-analyses.

Qualitative approaches can be used to assess a study from a process perspective: process evaluations, partly using qualitative or mixed methods, have proved useful to identify gaps in the design and conduct, as well as strengths and limitations of studies. A recent systematic review by Lazo-Porras and colleagues [6] highlights examples including qualitative methods for NTDs such as onchocerciasis. We conducted a qualitative assessment of the planning and initiation phase of the Gojjam Lymphoedema Best Practice Trial (GoLBeT) RCT [7], testing a simple intervention package for management of podoconiosis. Using content analysis of emails and reports, and interviews with the trial team, distinct steps between trial inception and recruitment were identified, along with elements that were seen as crucial to the successful setup of the trial in this setting.

Approaches to consult patients and their communities in the informed consent process using qualitative methods are not uncommon, and a number of examples and guidelines exist [8]. Some approaches might be better suited to NTDs: Rapid Ethical Assessment (REA) procedures are a type of qualitative intervention to ‘map the ethical terrain’ of a research setting by consulting communities on aspects of the study protocol, typically recruitment and informed consent procedures [9]. They can be valuable instruments particularly in areas that are difficult to access, or where a study team is less experienced; examples include REAs supporting podoconiosis studies in Cameroon [10] and Ethiopia [11]. REAs can be conducted within weeks and are inexpensive [9]; they have been shown to improve the quality of RCTs by boosting consent comprehension, the quality of the consent process and study recruitment and retention rates [12].

Other approaches have looked at acceptability of an intervention, such as a drug regimen, to patients and HCWs. Krentel and colleagues [13] embedded a mixed methods study in a community-based safety study assessing a new three-drug regimen for treatment of lymphatic filariasis. Maintz and colleagues [14] used comparable methods to assess the feasibility and acceptance of single-dose liposomal amphotericin B for the treatment of visceral leishmaniasis. Stringer and colleagues (2021) [15] have detailed the assessment of patients’ experiences and perspectives, patient-reported outcomes within the TB PRACTECAL trial. Of the studies that involved patients, HCWs, and researchers in the definition of study endpoints and the establishment of Core Outcome Sets, often within mixed methods designs [16], few are on NTDs [17].

## Strengths, limitations, and challenges

The suggested mixed methods approaches can yield information that could not be obtained otherwise: they can enrich DTA studies and RCTs for NTDs by characterizing the study process and target settings, by ensuring that the recruitment and informed consent procedures, interventions, and outcomes are understandable and acceptable to the target population, and by providing estimates for better grounded assumptions and parameters. Many of the study designs are highly participatory; instead of being removed from the research process, study participants are active and visible parts of it. The qualitative nature of these embedded studies might consist of focused questions, seeking specific information; or of very broad exploratory questions, seeking respondents’ preferences, views, and stories. In either case, studies benefit from valid and accepted methodologies for qualitative data analysis, such as thematic [18] or framework analysis [19], independent of study size, and contribute to engendering “*action intending to save lives and reduce suffering”* [20].

While qualitative studies’ findings often have limited generalizability and representativeness in comparison to quantitative studies [21], they are, by focusing on understanding a specific setting, capable of answering highly contextual questions. Studies often use purposive sampling approaches resulting in much smaller sample sizes than the parent study they are embedded in. Sample size should be guided by considerations related to theoretical data saturation (a criterion for discontinuing data collection and/or analysis) [22]. The research team needs to be open to using mixed methods and should include relevant expertise, such as team members who have been trained in qualitative research methods, as well as methods to integrate qualitative and quantitative data, which can be challenging [23]. Moreover, it is important that studies are conducted in a transparent manner, with respect for the privacy of study participants, and fully in compliance with ethical considerations, following a detailed protocol and rigorous consent procedures.

Researchers based at the University of York have provided an extensive toolkit for studies-within-a-trial (SWATs) [24], methodological studies (which can be qualitative) aiming at generating new knowledge to improve the design and delivery of trials. Funders and journals are usually more familiar with one or the other study type, and studies or proposals using both quantitative and qualitative data might require reviewers, editors, and decision-makers to be familiar with both research approaches, in addition to subject-specific knowledge, which may pose a challenge. Lastly, timelines are often prohibitive to seeking separate ethical approval for such comparatively small studies (perceived in relation to the parent study), in particular if urgent action is needed. The availability of examples and template protocols for such embedded studies (similar to templates available for trials [25]), and the integration of ‘placeholder’ components in the parent study protocol to be submitted for ethical approval would help research groups at the study planning phase. Prior to onset of the embedded study, an amendment could then provide the required level of detail regarding this component; overall, this could help accelerate the process without compromising quality, and, importantly, facilitate publication.

## Conclusion

Engaging researchers, HCWs, and communities in the research process *via* methodologically rigorous qualitative studies embedded in trials evaluating medical innovations can help to address crucial aspects about the design of these trials that will ensure swift uptake of research outcomes. This is particularly relevant for studies of NTDs, where funding is limited and opportunities are few, and which may require tailored approaches for vulnerable, disadvantaged populations in often-complex implementation contexts. This requires consulting researchers, HCWs, and the communities where the study will be done. Such mixed methods studies have been shown to be useful and affordable parts of their ‘parent’ studies; more examples and methodological guidance are needed for such highly participatory study types for NTDs, where they can meaningfully complement other implementation research efforts [26].
